# Supplementary material for: Mechanistic classification and benchmarking of polyolefin depolymerization over silica-alumina-based catalysts
Source: Nat Commun. 2022 Aug 17;13:4850. doi: 10.1038/s41467-022-32563-y (PMC9385622; doi:10.1038/s41467-022-32563-y)
Supplement: Supplementary file 1 — Supplementary Information [file 41467_2022_32563_MOESM1_ESM.pdf]

Supplementary Information

Mechanistic classification and benchmarking of polyolefin depolymerization over silica-alumina-based catalysts

Wei-Tse Lee[1], Antoine van Muyden[1], Felix D. Bobbink[1], Mounir D. Mensi[1], Jed R. Carullo[1], Paul J. Dyson[1,*]

[1]Institute of Chemical Sciences and Engineering, École Polytechnique Fédérale de Lausanne (EPFL), Lausanne, Switzerland.
[*]Corresponding author: Paul J. Dyson (paul.dyson@epfl.ch)

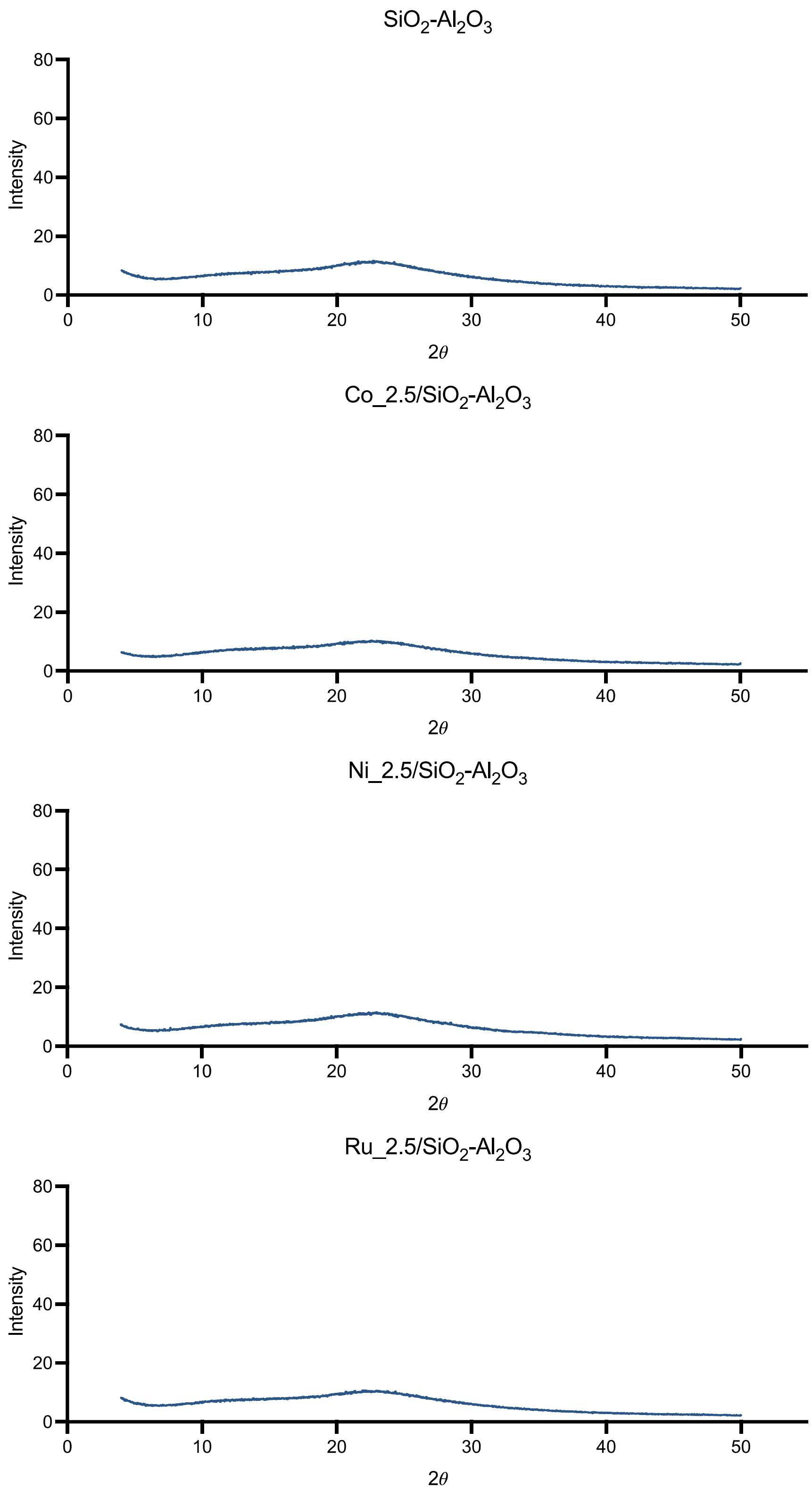

Supplementary Figure 1 Powder X-ray of SiO_2-Al_2O_3-based catalysts. Measurements of SiO_2-Al_2O_3, Co/SiO_2-Al_2O_3, Ni/SiO_2-Al_2O_3 and Ru/SiO_2-Al_2O_3 with 2θ = 4-50 degree. Source data are provided as a Source Data file.

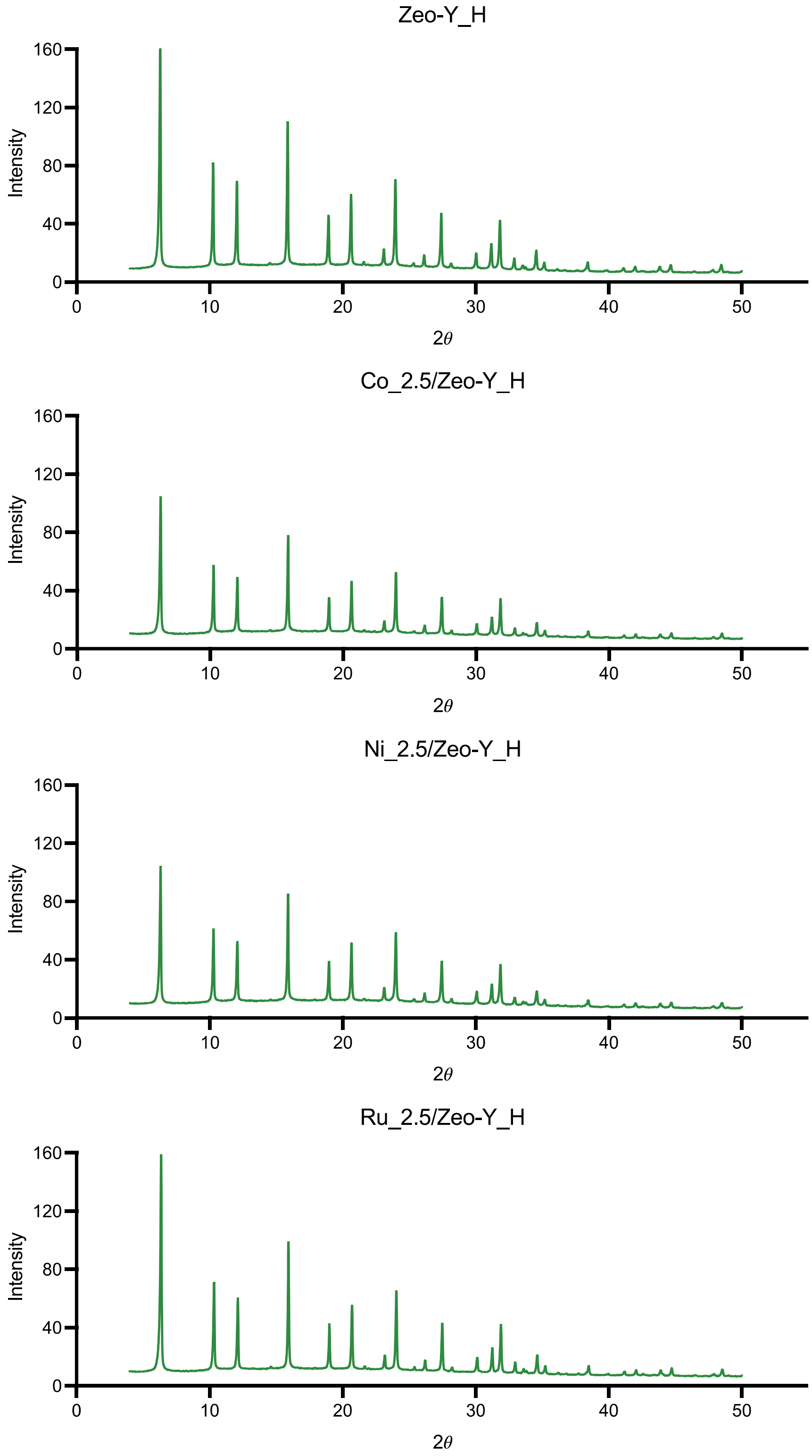

Supplementary Figure 2 Powder X-ray of Zeo-Y_H-based catalysts. Measurements of Zeo-Y_H, Co/Zeo-Y_H, Ni/Zeo-Y_H and Ru/Zeo-Y_H with 2θ = 4-50 degree. Source data are provided as a Source Data file.

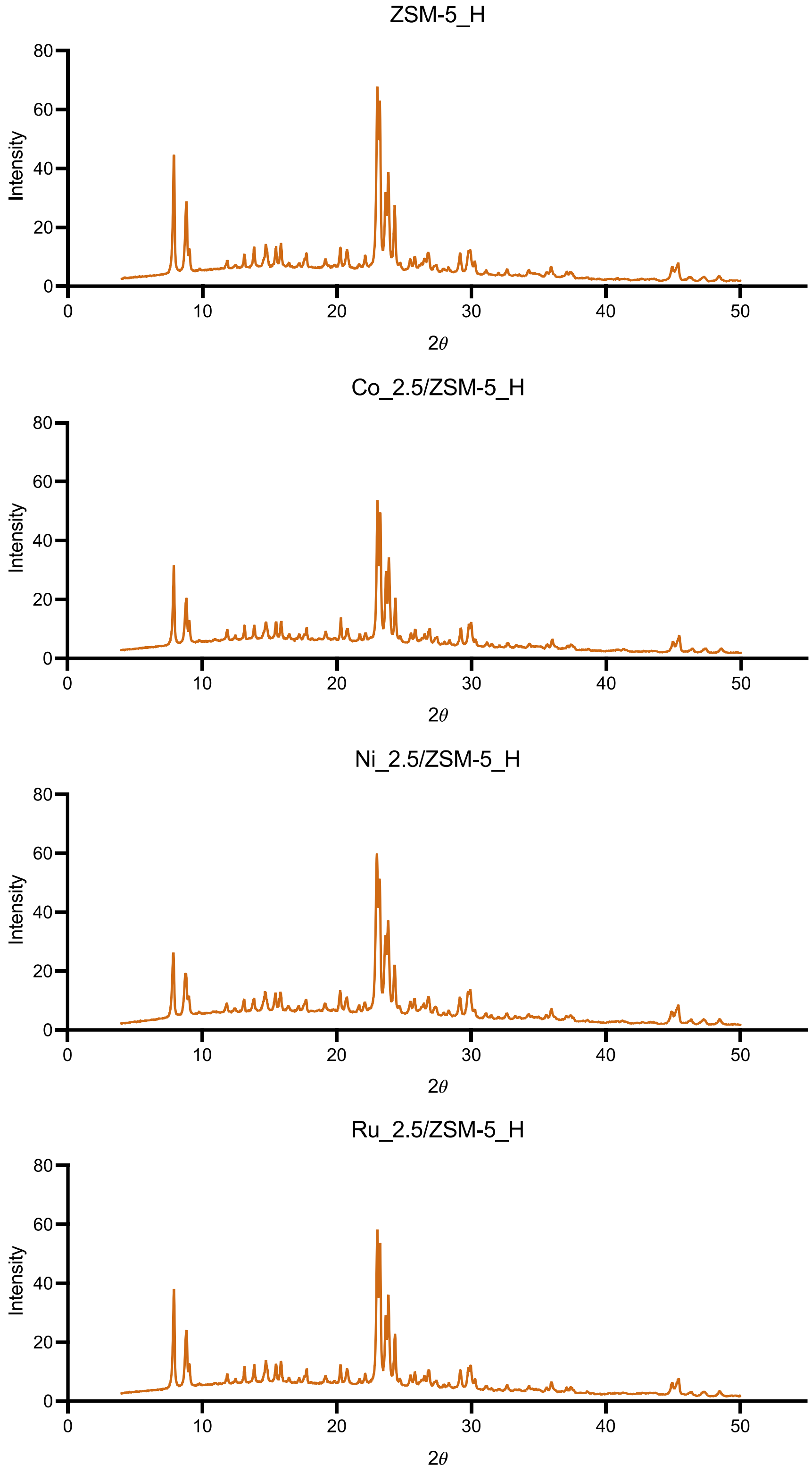

Supplementary Figure 3 Powder X-ray of ZSM-5_H-based catalysts. Measurements of ZSM-5_H, Co/ZSM-5_H, Ni/ZSM-5_H and Ru/ZSM-5_H with 2θ = 4-50 degree. Source data are provided as a Source Data file.

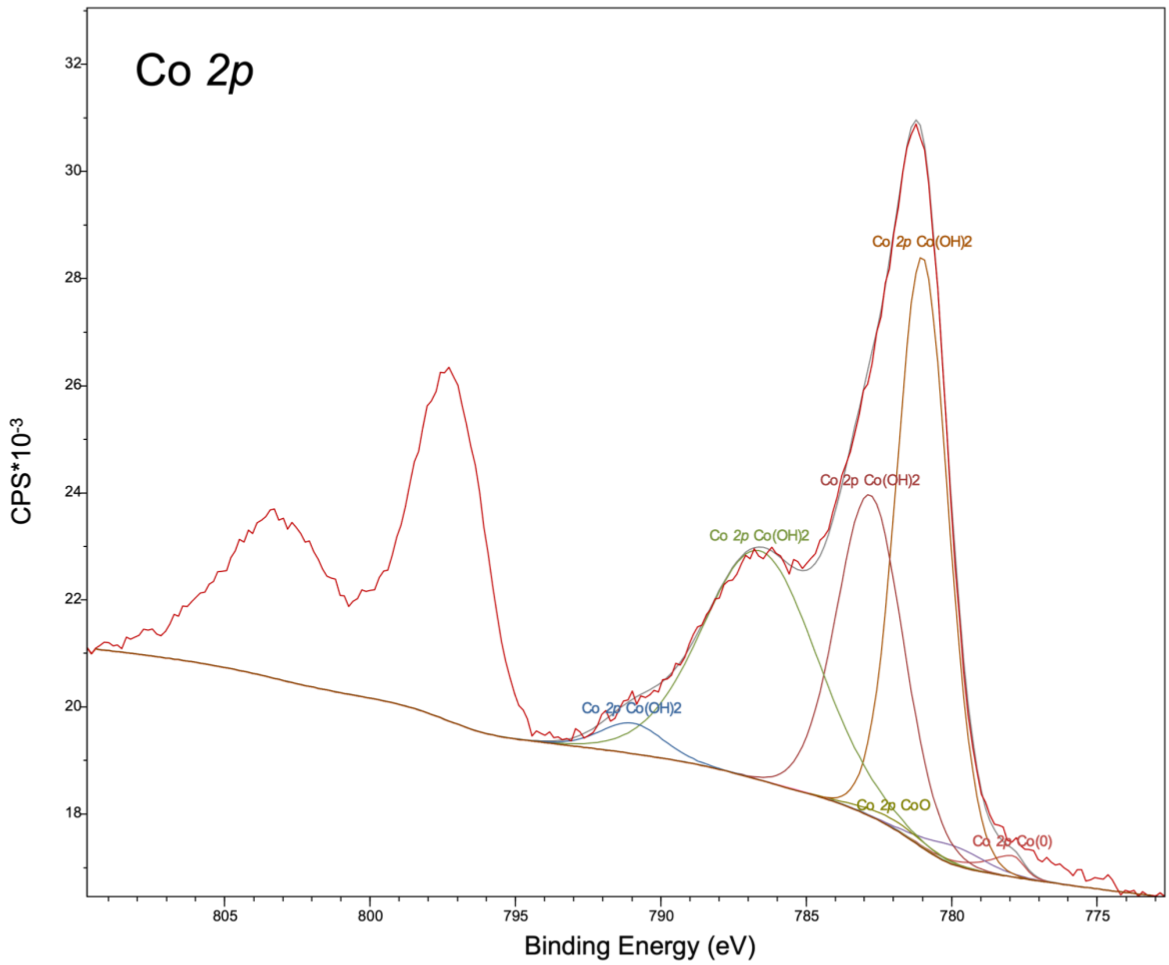

Supplementary Figure 4 Curve fitted XPS analysis of Co *2p* region of Co/ZSM-5_H catalyst. Fitting according to Mark Biesinger et al.[1] Source data are provided as a Source Data file.

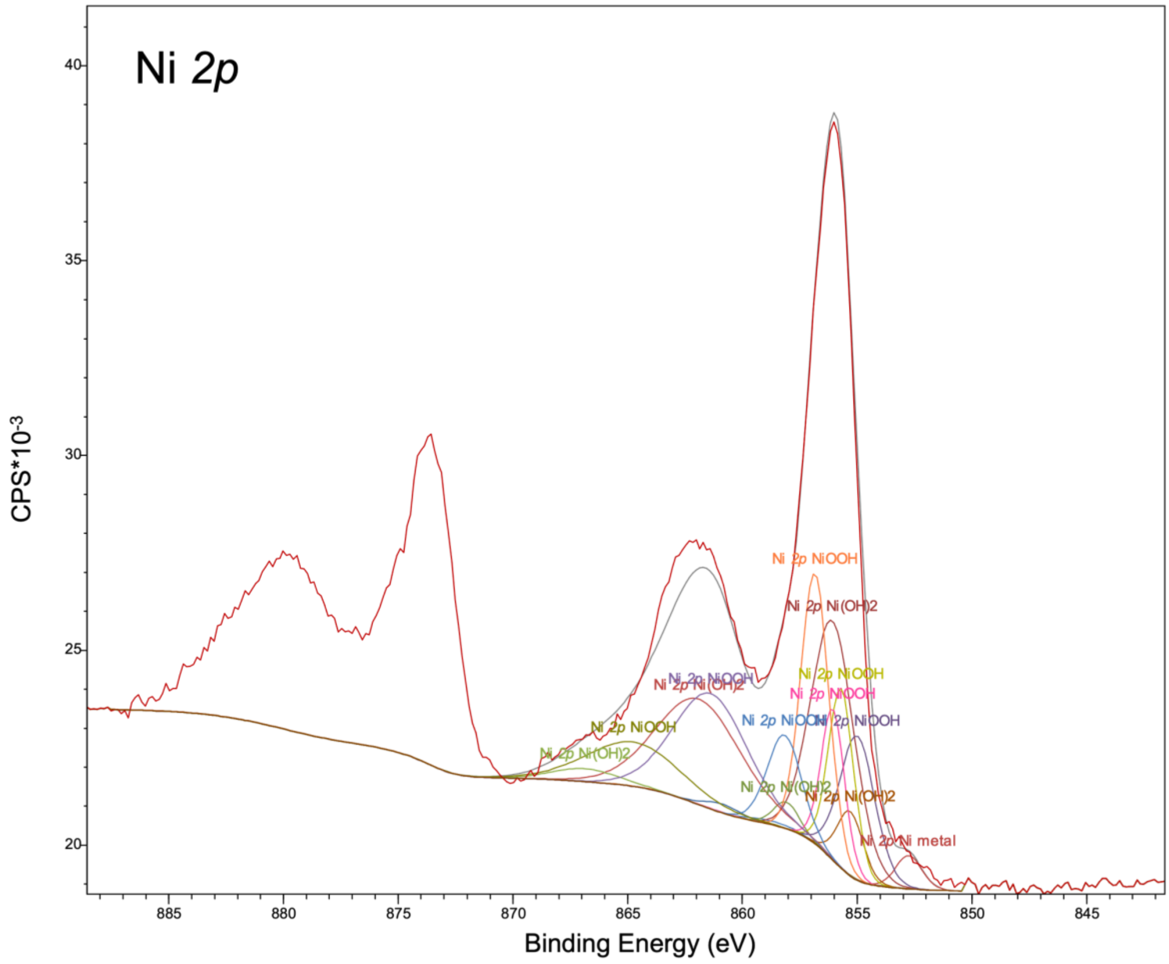

Supplementary Figure 5 Curve fitted XPS analysis of Ni *2p* region of Ni/ZSM-5_H catalyst. Fitting according to Mark Biesinger et al.[1] Source data are provided as a Source Data file.

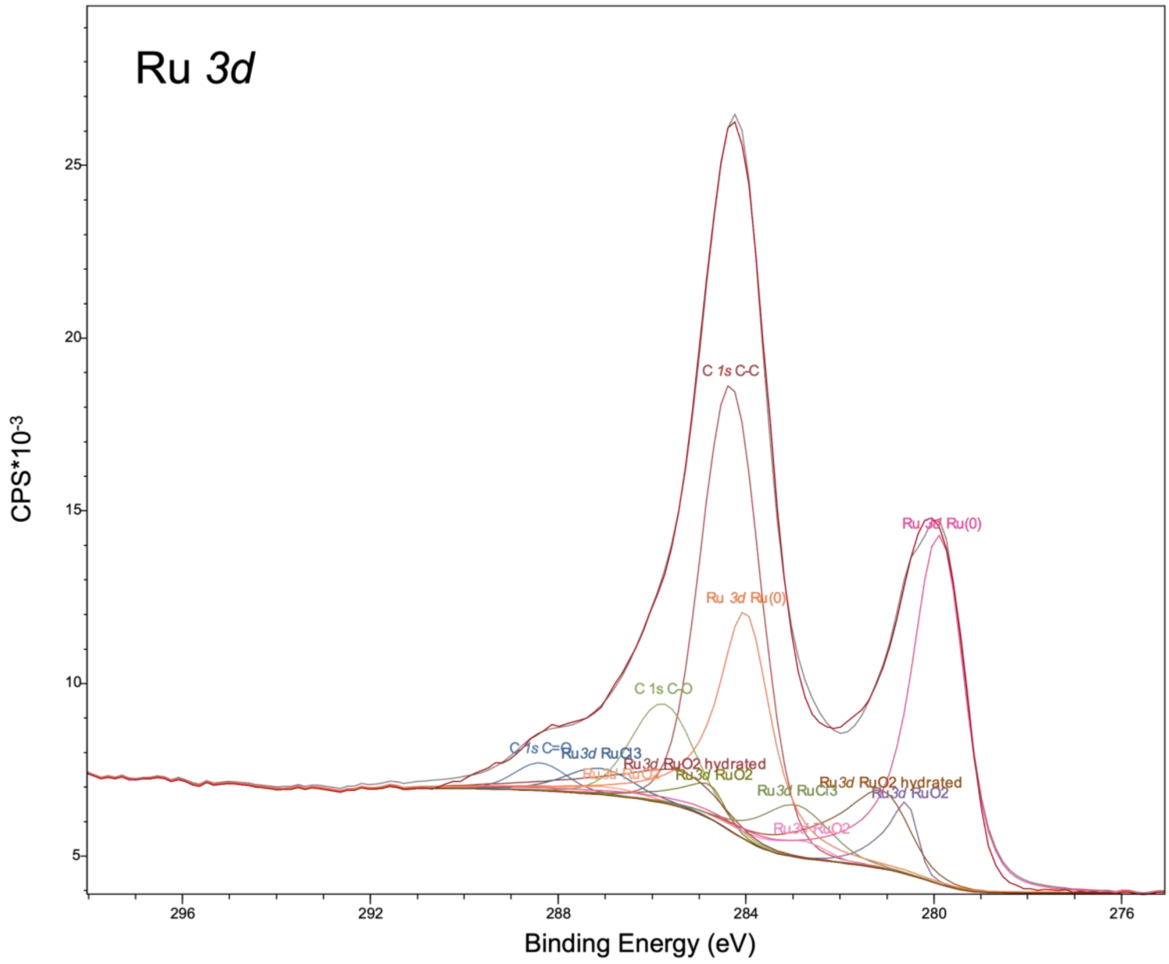

Supplementary Figure 6 Curve fitted XPS analysis of Ru *3d* and C *1s* region of Ru/ZSM-5_H catalyst. Fitting according to David J. Morgan.[2] Source data are provided as a Source Data file.

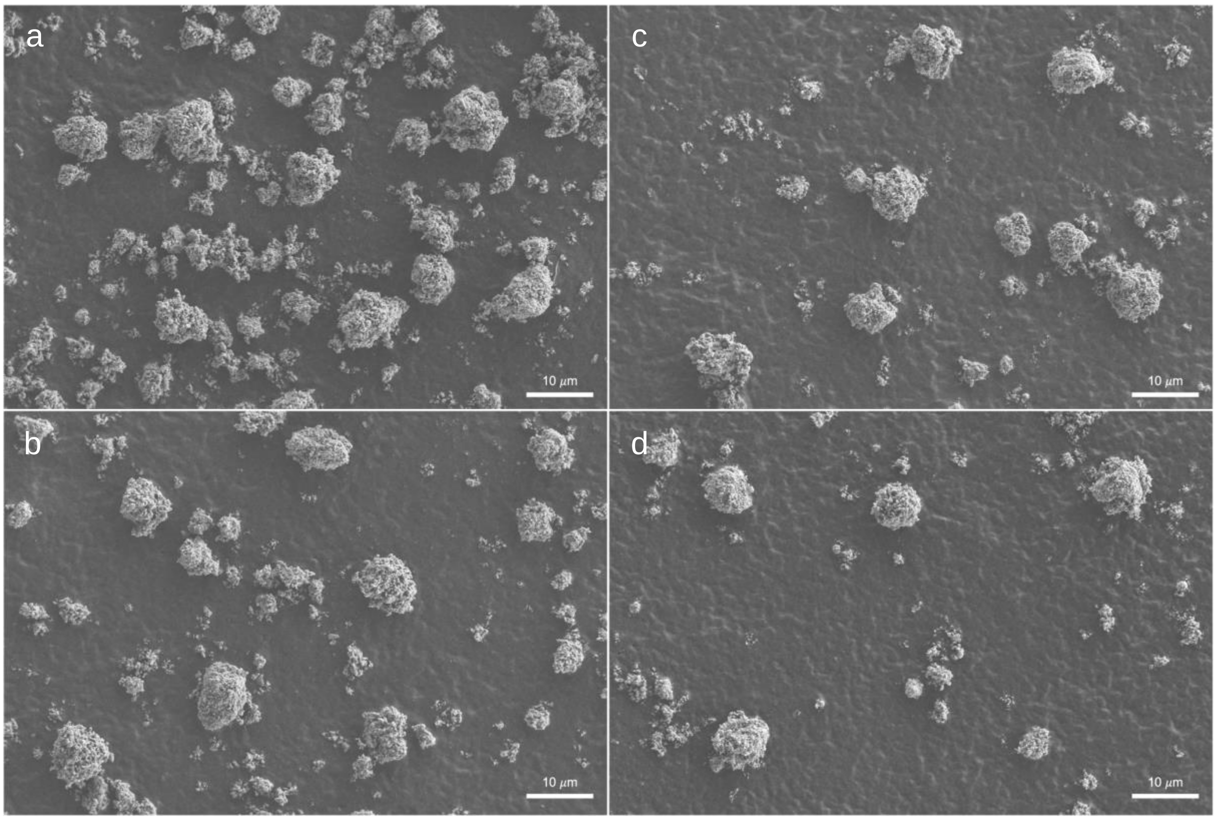

Supplementary Figure 7 SEM images (scale bar = 10 μm) showing the particle distributions. a ZSM-5_H (6.4-9.4 μm), **b** Co/ZSM-5_H (6.3-9.7 μm), **c** Ni/ZSM-5_H (6.3-8.6 μm) and **d** Ru/ZSM-5_H (6.0-10.4 μm).

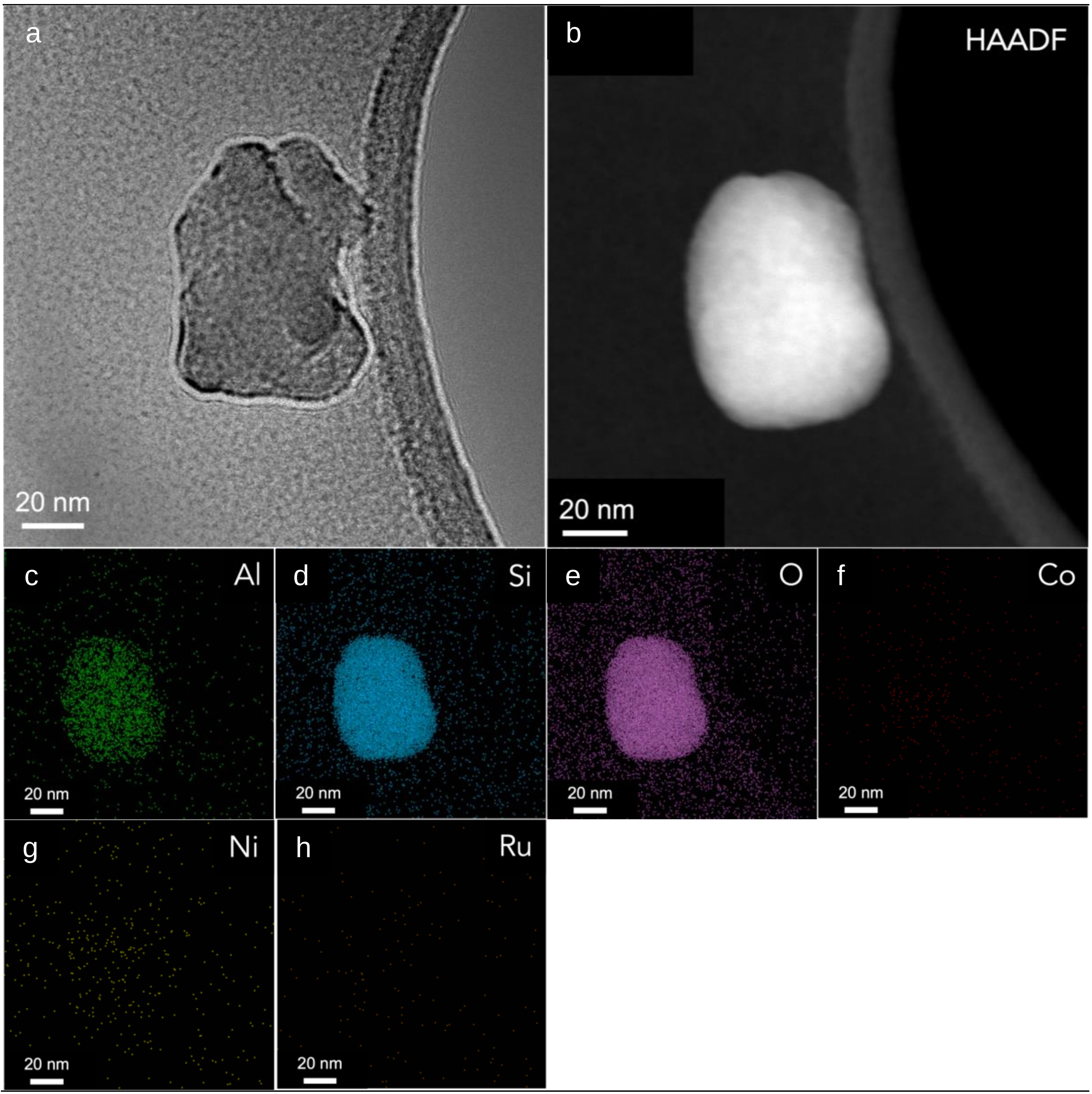

Supplementary Figure 8 Electron microscopy images of ZSM-5_H. TEM bright-field image (scale bar = 20 nm) of **a** ZSM-5_H. HAADF image (scale bar = 20 nm) of **b** ZSM-5_H. STEM-EDS elemental mapping of **c** Al, **d** Si, **e** O, **f** Co, **g** Ni and **h** Ru.

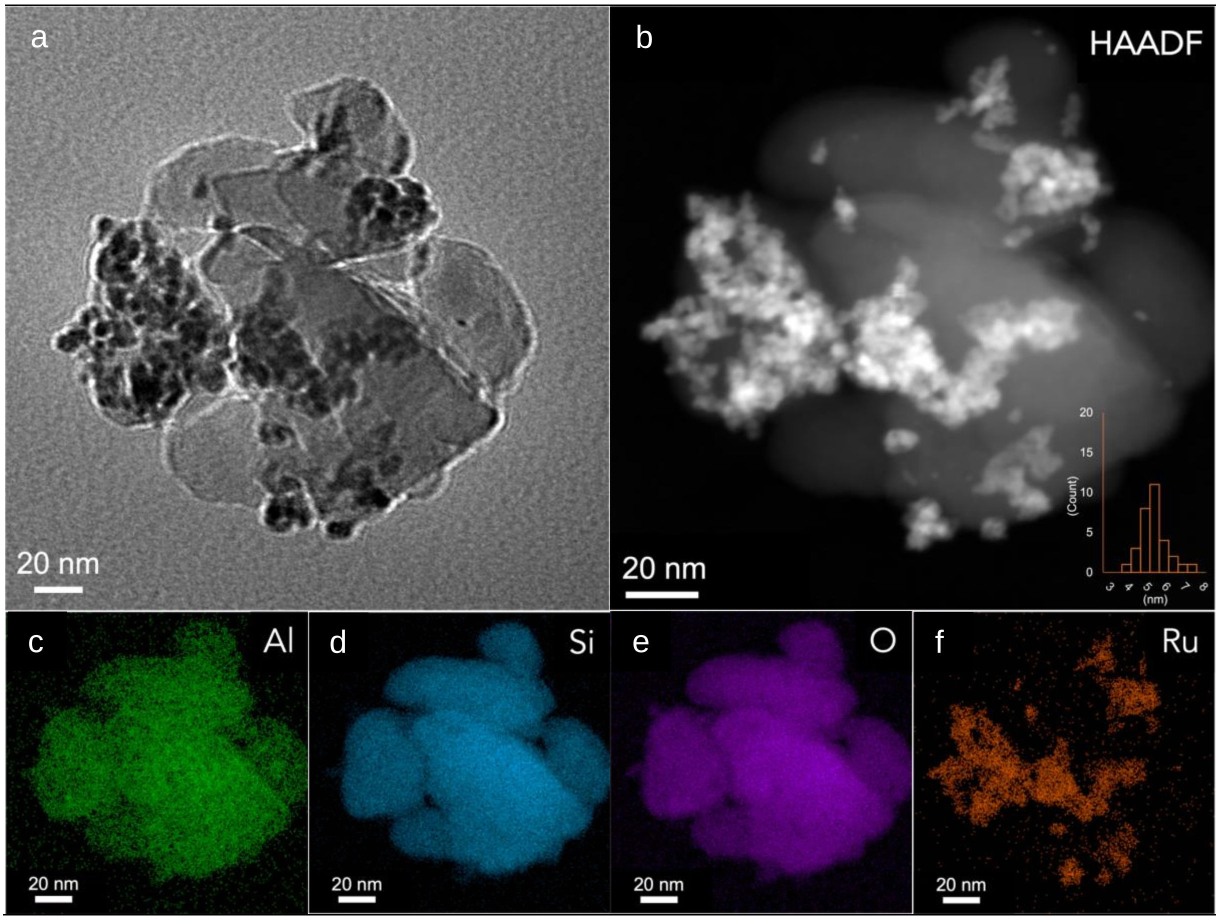

Supplementary Figure 9 Electron microscopy images of Ru/ZSM-5_H. TEM bright-field image (scale bar = 20 nm) of **a** Ru/ZSM-5_H. HAADF image (scale bar = 20 nm) of **b** Ru/ZSM-5_H with Ru NP distributions = 5.4±0.8 nm. STEM-EDS elemental mapping of **c** Al, **d** Si, **e** O and **f** Ru.

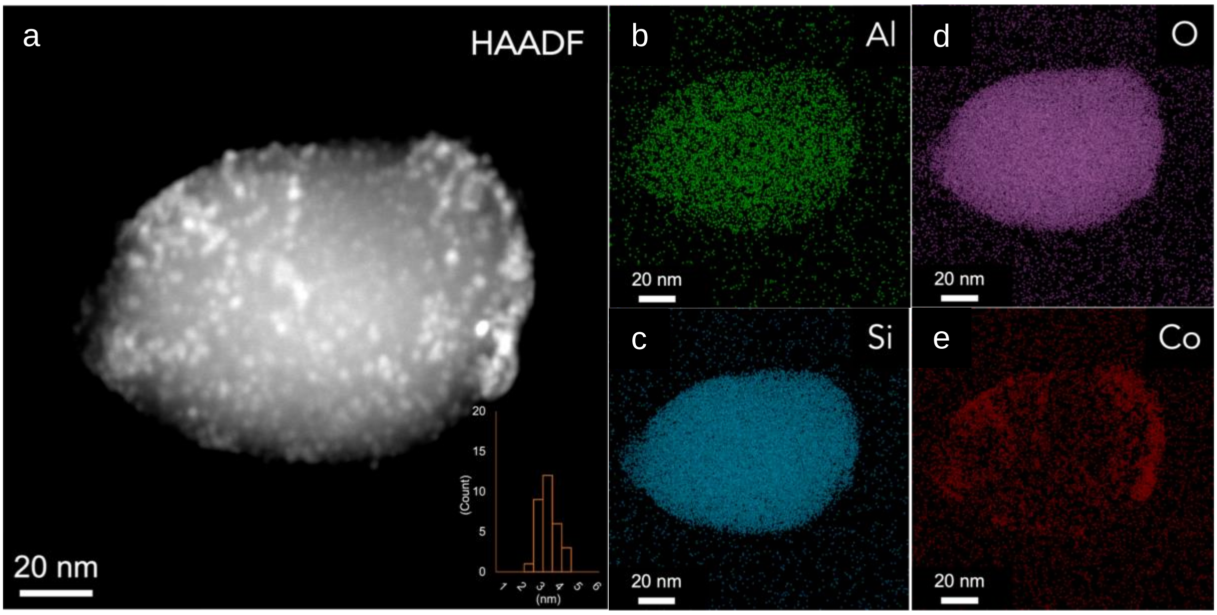

Supplementary Figure 10 Electron microscopy images of Co/ZSM-5_H. HAADF image (scale bar = 20 nm) of **a** Co/ZSM-5_H with Co NP distributions = 3.6±0.5 nm. STEM-EDS elemental mapping of **b** Al, **c** Si, **d** O and **e** Co.

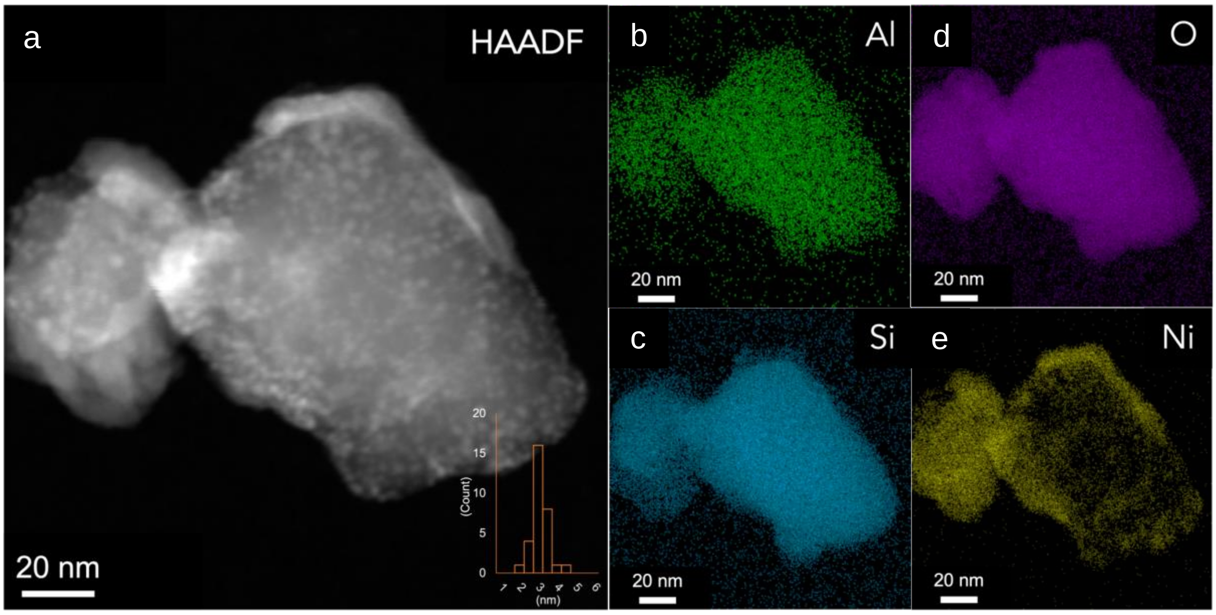

Supplementary Figure 11 Electron microscopy images of Ni/ZSM-5_H. HAADF image (scale bar = 20 nm) of **a** Ni/ZSM-5_H with Ni NP distributions = 3.1±0.4 nm. STEM-EDS elemental mapping of **b** Al, **c** Si, **d** O and **e** Ni.

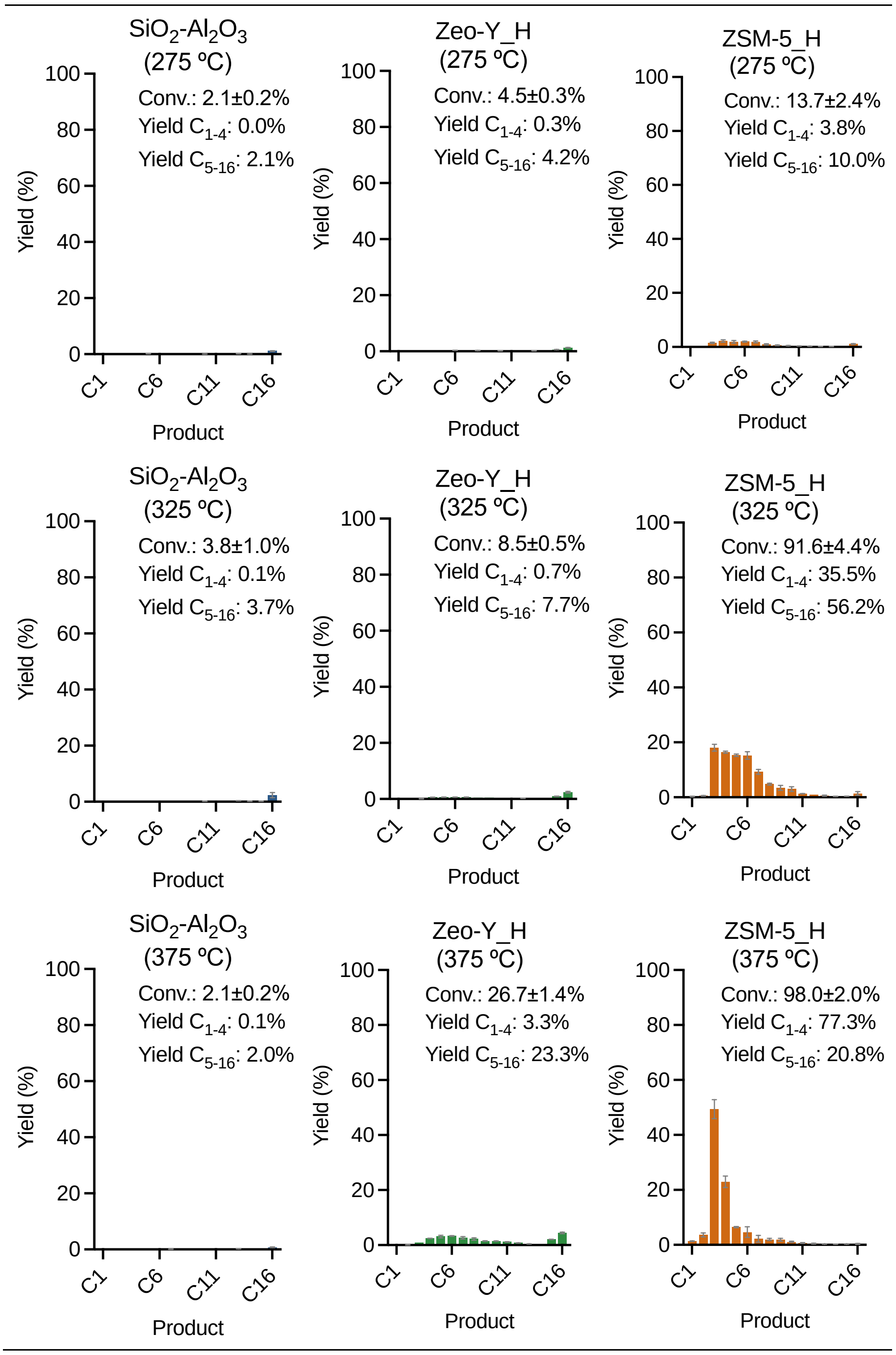

Supplementary Figure 12 Product distributions after nC_{16} (1.59 g) deconstructions. In the presence of unmodified silica-alumina catalysts (0.1 g): SiO_2-Al_2O_3, Zeo-Y_H and ZSM-5_H, 45 bar H_2, 2 hrs. Note that nC_{12} signal originated from the addition as an internal standard has been suppressed, and the yield of C_{12} products was derived from the nC_{16} substrate. Error bars = standard deviation. Source data are provided as a Source Data file.

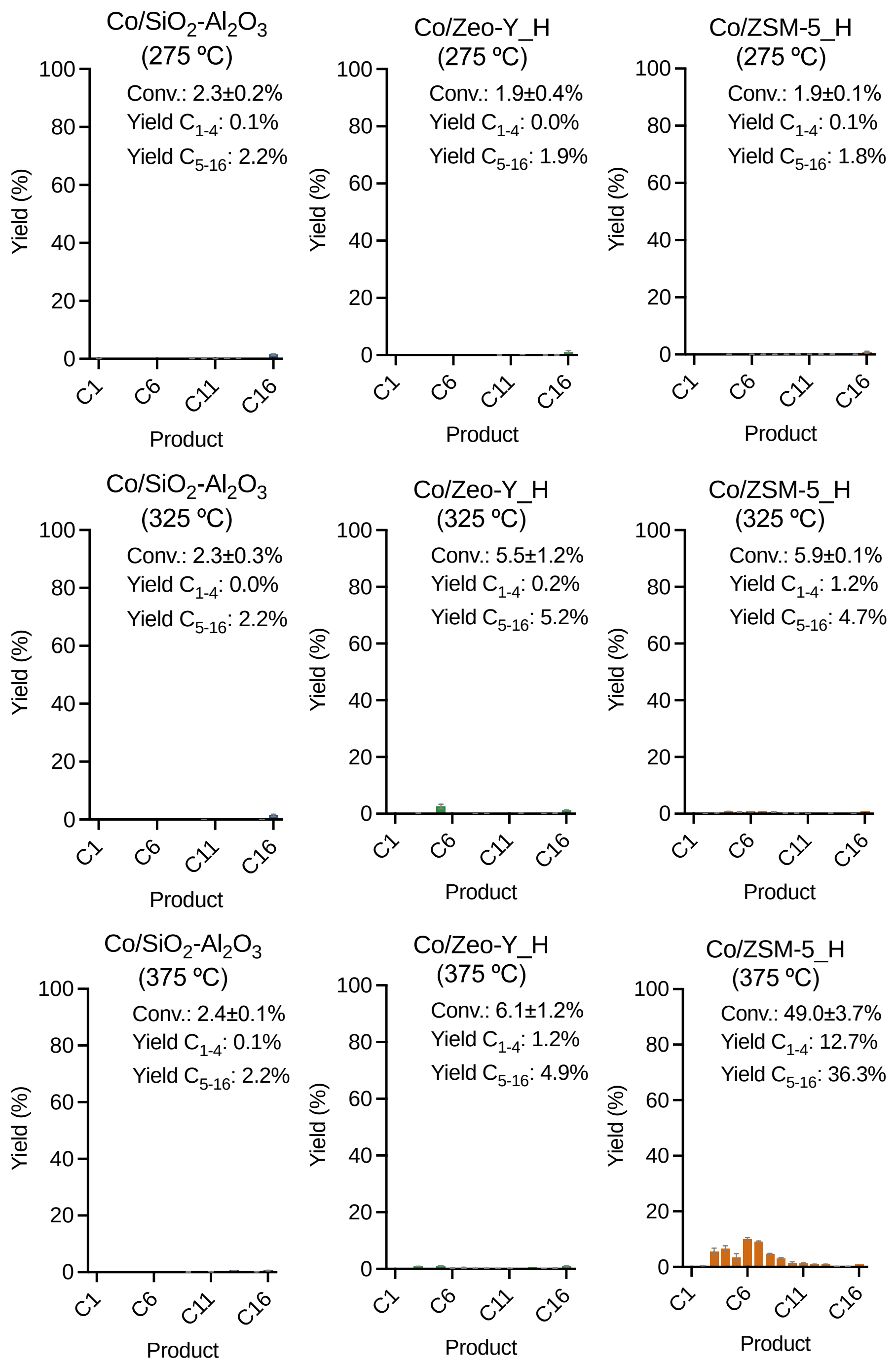

Supplementary Figure 13 Product distributions after nC_{16} (1.59 g) deconstructions. In the presence of Co-modified catalysts (0.1 g, metal loading = 2.5 wt%): Co/SiO_2-Al_2O_3, Co/Zeo-Y_H and Co/ZSM-5_H, 45 bar H_2, 2 hrs. Note that nC_{12} signal originated from the addition as an internal standard has been suppressed, and the yield of C_{12} products was derived from the nC_{16} substrate. Error bars = standard deviation. Source data are provided as a Source Data file.

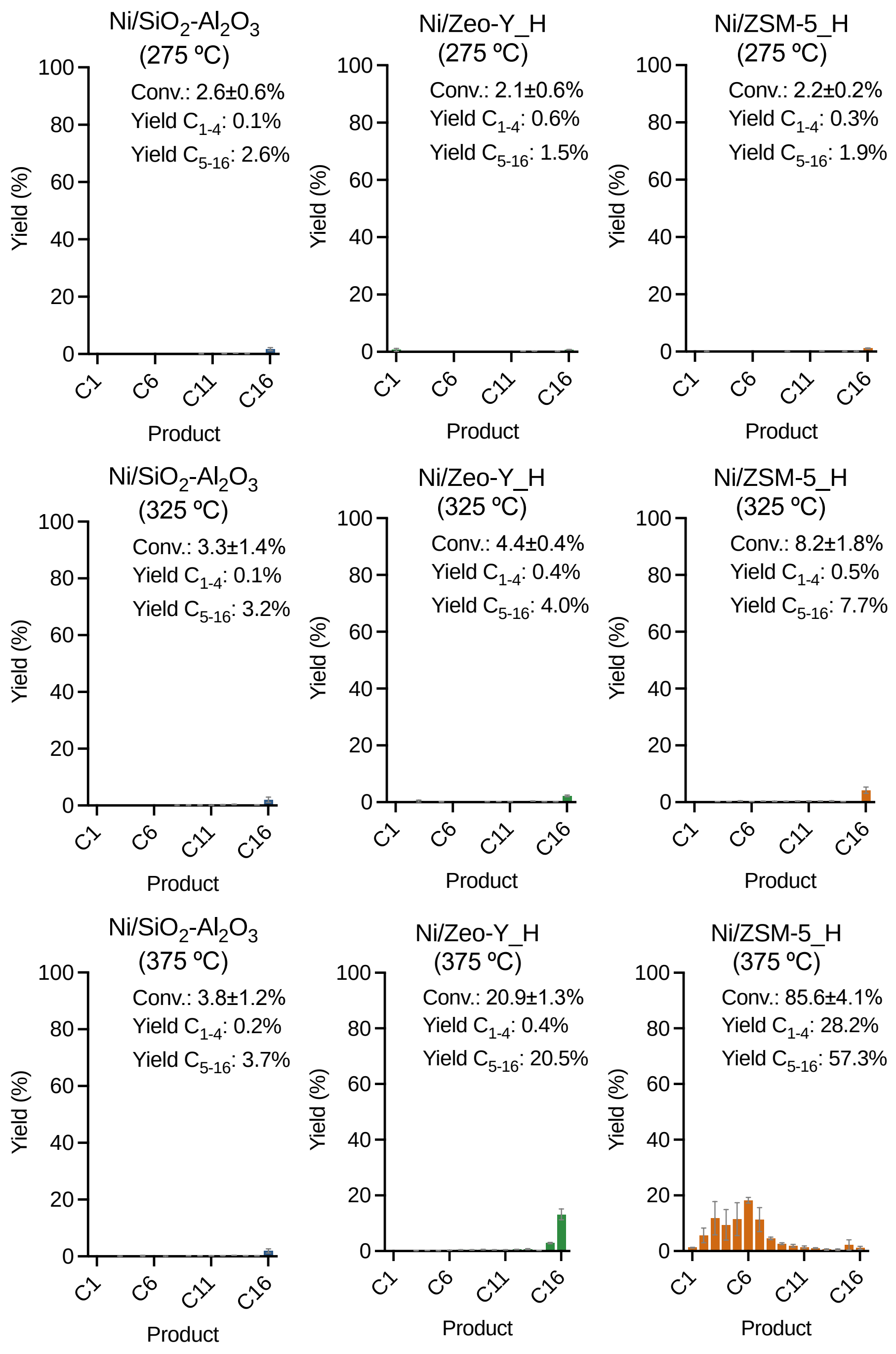

Supplementary Figure 14 Product distributions after nC_{16} (1.59 g) deconstructions. In the presence of Ni-modified catalysts (0.1 g, metal loading = 2.5 wt%): Ni/SiO_2-Al_2O_3, Ni/Zeo-Y_H and Ni/ZSM-5_H, 45 bar H_2, 2 hrs. Note that nC_{12} signal originated from the addition as an internal standard has been suppressed, and the yield of C_{12} products was derived from the nC_{16} substrate. Error bars = standard deviation. Source data are provided as a Source Data file.

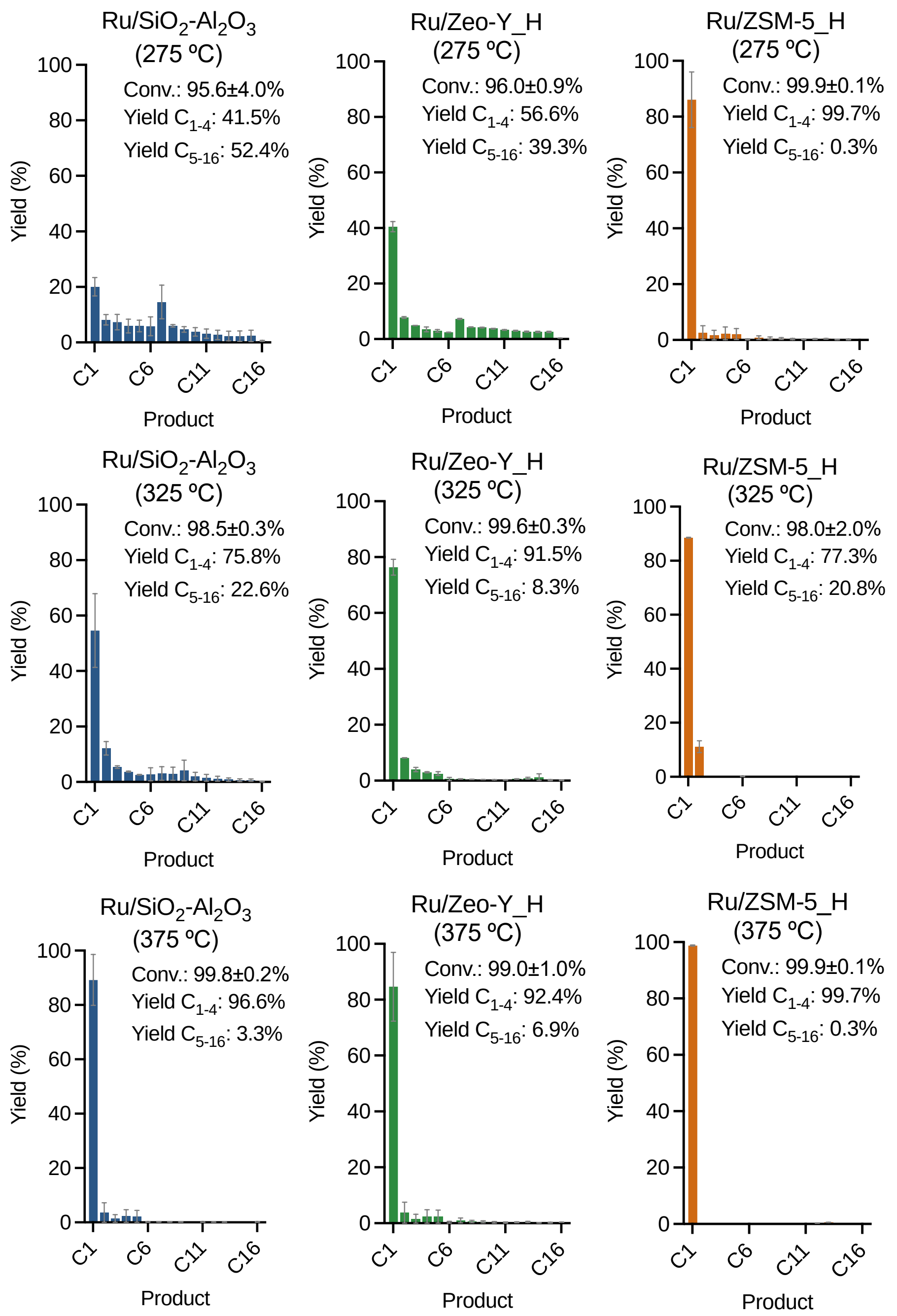

Supplementary Figure 15 Product distributions after nC_{16} (1.59 g) deconstructions. In the presence of Ru-modified catalysts (0.1 g, metal loading = 2.5 wt%): Ru/SiO_2-Al_2O_3, Ru/Zeo-Y_H and Ru/ZSM-5_H, 45 bar H_2, 2 hrs. Note that nC_{12} signal originated from the addition as an internal standard has been suppressed, and the yield of C_{12} products was derived from the nC_{16} substrate. Error bars = standard deviation. Source data are provided as a Source Data file.

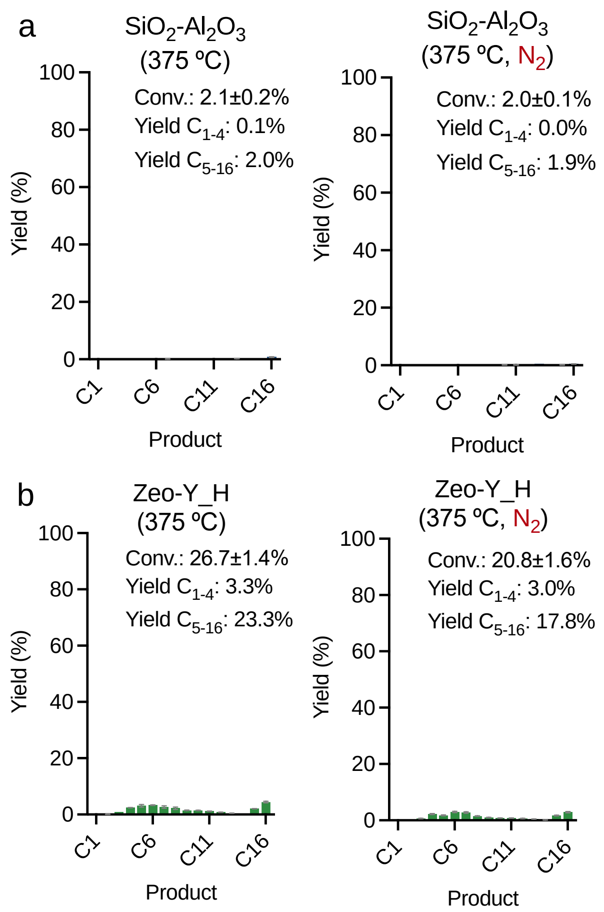

Supplementary Figure 16 Product distributions after nC_{16} (1.59 g) deconstructions. In the presence of **a** SiO_2-Al_2O_3 (0.1 g) and **b** Zeo-Y_H (0.1 g) under 45 bar H_2 and N_2 at 375 ºC, 2 hrs. Note that nC_{12} signal originated from the addition as an internal standard has been suppressed, and the yield of C_{12} products was derived from the nC_{16} substrate. Error bars = standard deviation. Source data are provided as a Source Data file.

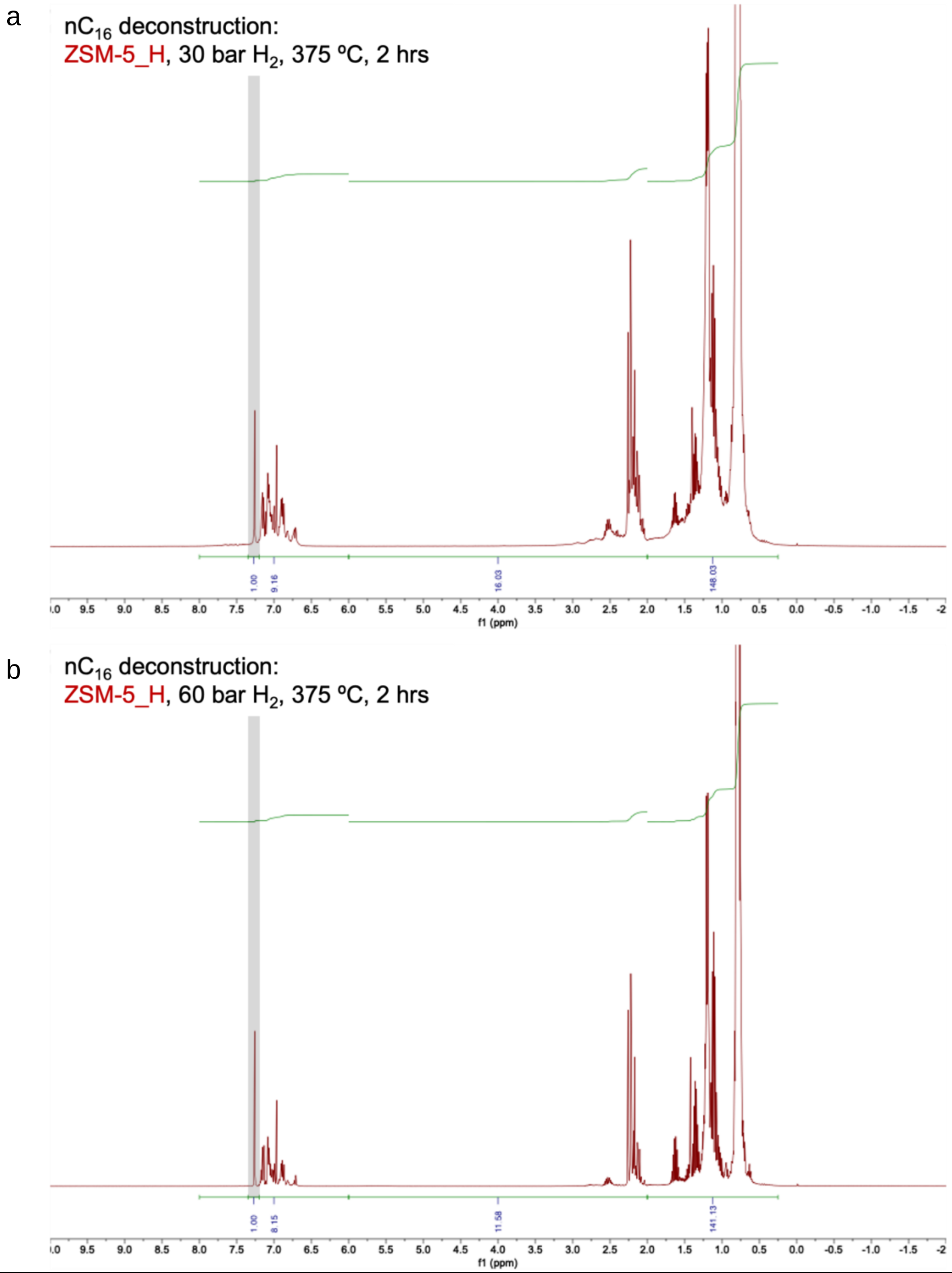

Supplementary Figure 17 ^{1}H NMR spectra of liquid products after nC_{16} (1.59 g) deconstruction. In the presence of ZSM-5_H (0.1 g) 375 ºC, 2 hrs under **a** 30 bar H_2, proton integration: 1.00, 9.16, 16.03, 148.03 = $CDCl_3$ (gray marked area, δ = 7.20-7.35), aromatic (δ = 6.0-8.0), unsaturated (δ = 2.0-6.0), saturated (δ = 0.25-2.0) and **b** 60 bar H_2, proton integration: 1.00, 8.15, 11.58, 141.13 = $CDCl_3$ (gray marked area, δ = 7.20-7.35), aromatic (δ = 6.0-8.0), unsaturated (δ = 2.0-6.0), saturated (δ = 0.25-2.0).

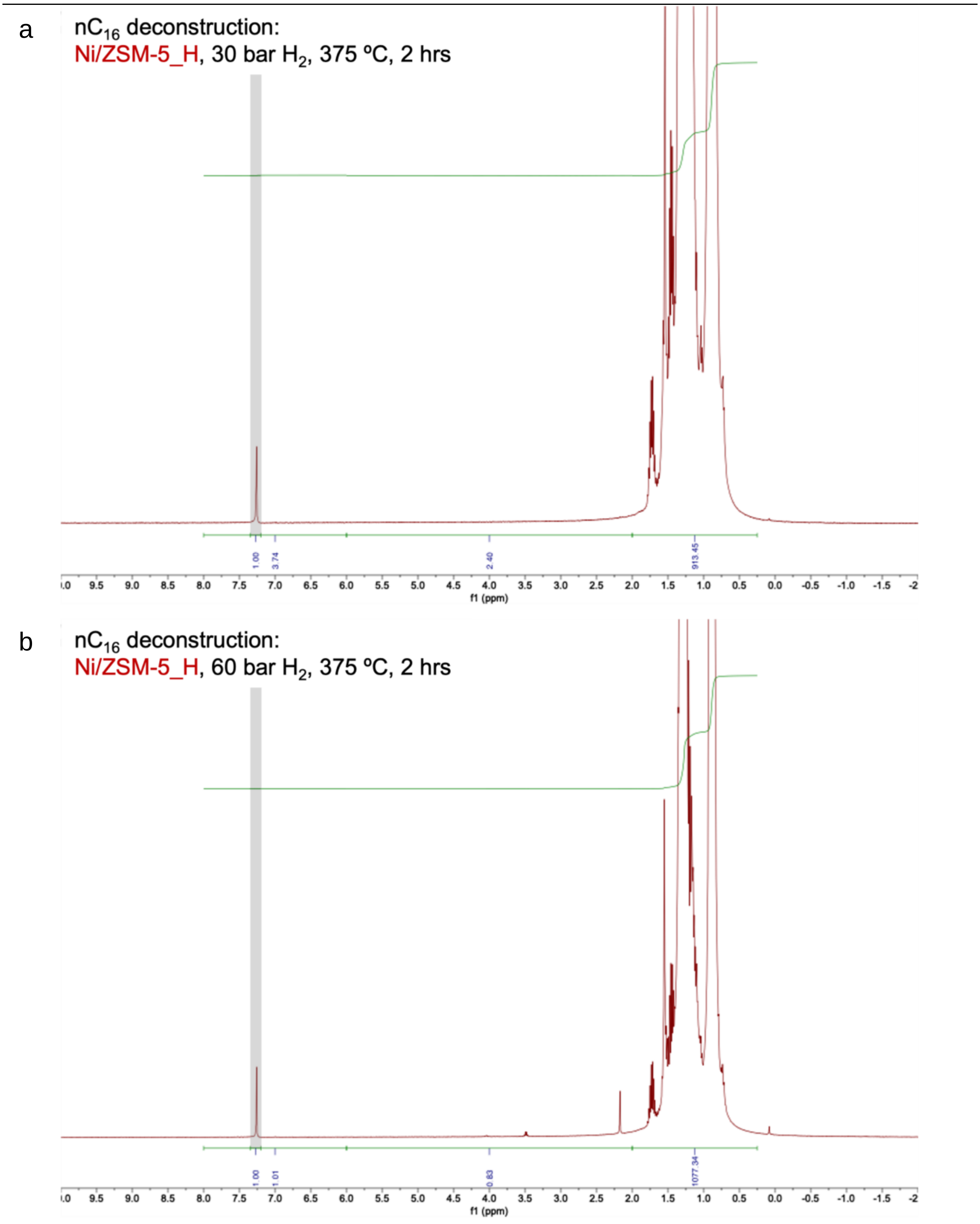

Supplementary Figure 18 ^{1}H NMR spectra of liquid products after nC_{16} (1.59 g) deconstruction. In the presence of Ni/ZSM-5_H (0.1 g) 375 ºC, 2 hrs under **a** 30 bar H_2, proton integration: 1.00, 3.74, 2.40, 913.45 = $CDCl_3$ (gray marked area, δ = 7.20-7.35), aromatic (δ = 6.0-8.0), unsaturated (δ = 2.0-6.0), saturated (δ = 0.25-2.0) and **b** 60 bar H_2, proton integration: 1.00, 1.01, 0.83, 1077.34 = $CDCl_3$ (gray marked area, δ = 7.20-7.35), aromatic (δ = 6.0-8.0), unsaturated (δ = 2.0-6.0), saturated (δ = 0.25-2.0).

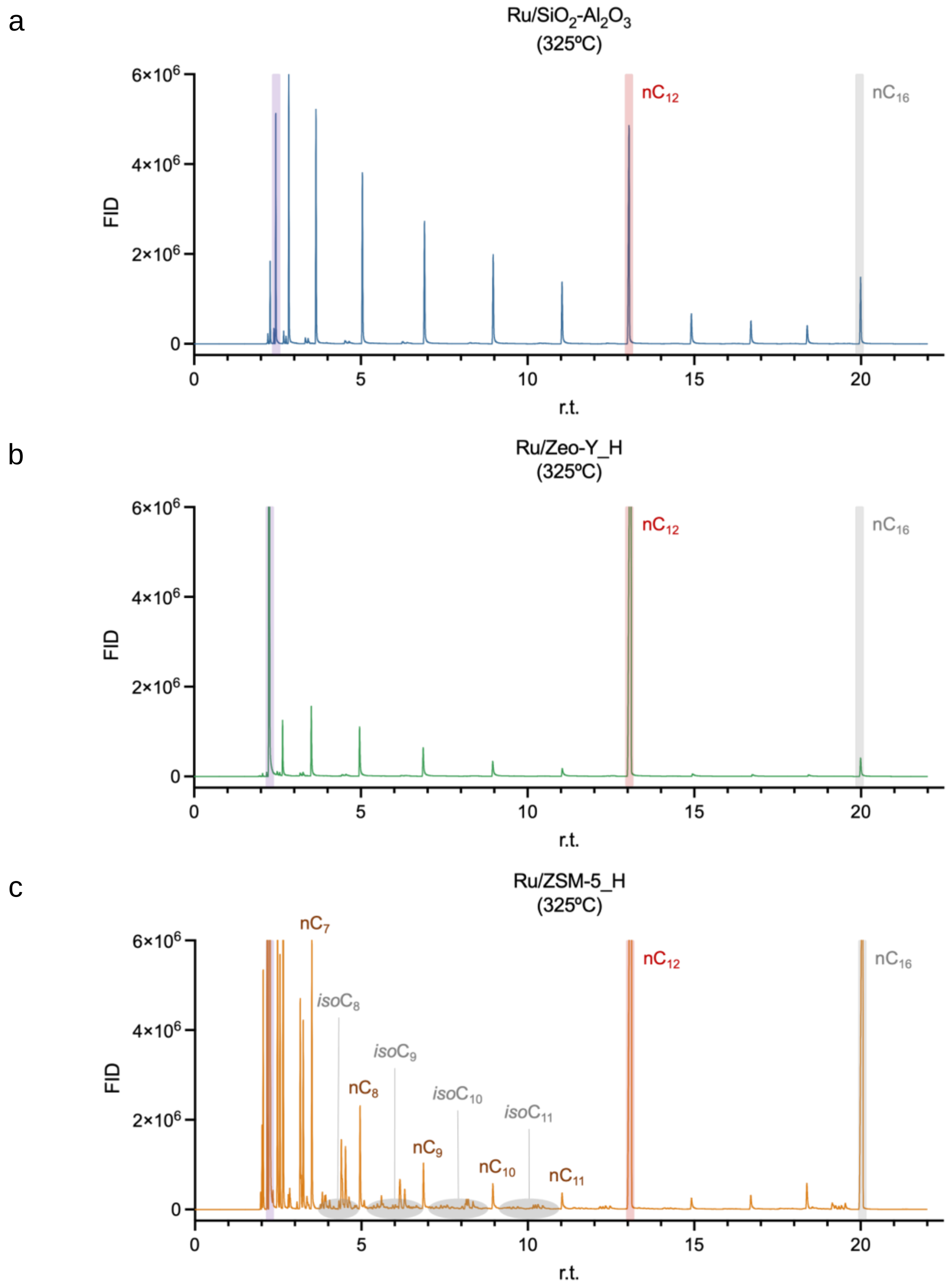

Supplementary Figure 19 GC-FID traces of liquid products after the nC_{16} (1.59 g) deconstruction. In the presence of **a** Ru/SiO_2-Al_2O_3 (0.1 g, metal loading = 2.5 wt%), **b** Ru/Zeo-Y_H (0.1 g, metal loading = 2.5 wt%) and **c** Ru/ZSM-5_H (0.1 g, metal loading = 2.5 wt%) under 45 bar H_2 at 325 ºC, 2 hrs. Note that the peaks with retention time ~2.7 and ~14.1 min were diethyl ether (solvent for the GC sample) and nC_{12} (internal standard), respectively.

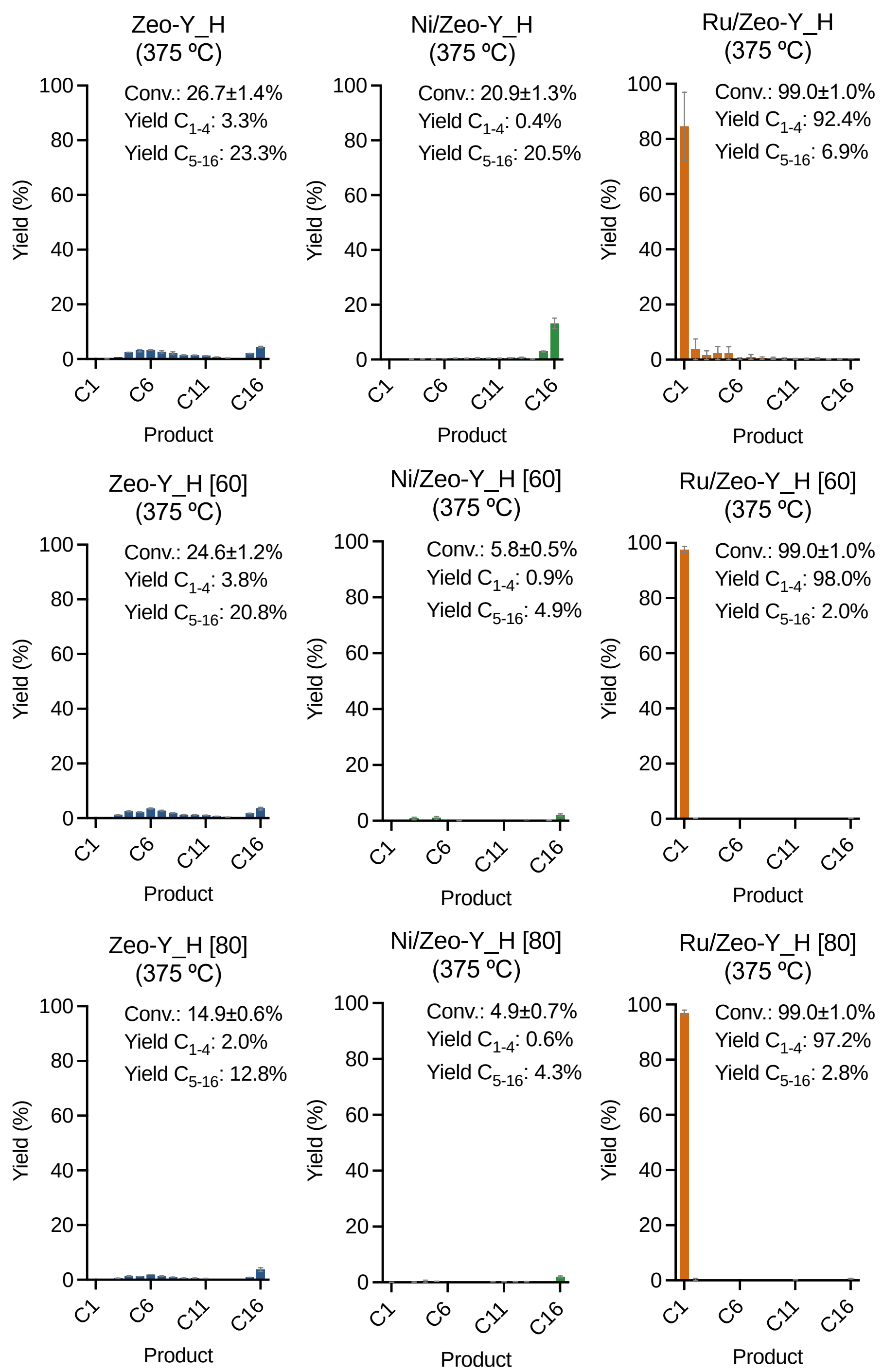

Supplementary Figure 20 Product distributions after nC_{16} (1.59 g) deconstructions. In the presence of Zeo-Y_H-based catalysts with various Si/Al ratios (0.1 g, metal loading = 2.5 wt%): Zeo-Y_H, Zeo-Y_H [60], Zeo-Y_H [80], Ni/Zeo-Y_H, Ni/Zeo-Y_H [60], Ni/Zeo-Y_H [80], Ru/Zeo-Y_H, Ru/Zeo-Y_H [60], and Ru/Zeo-Y_H [80], 45 bar H_2, 2 hrs. Note that nC_{12} signal originated from the addition as an internal standard has been suppressed, and the yield of C_{12} products herein is derived from the nC_{16} substrate. Error bars = standard deviation. Source data are provided as a Source Data file.

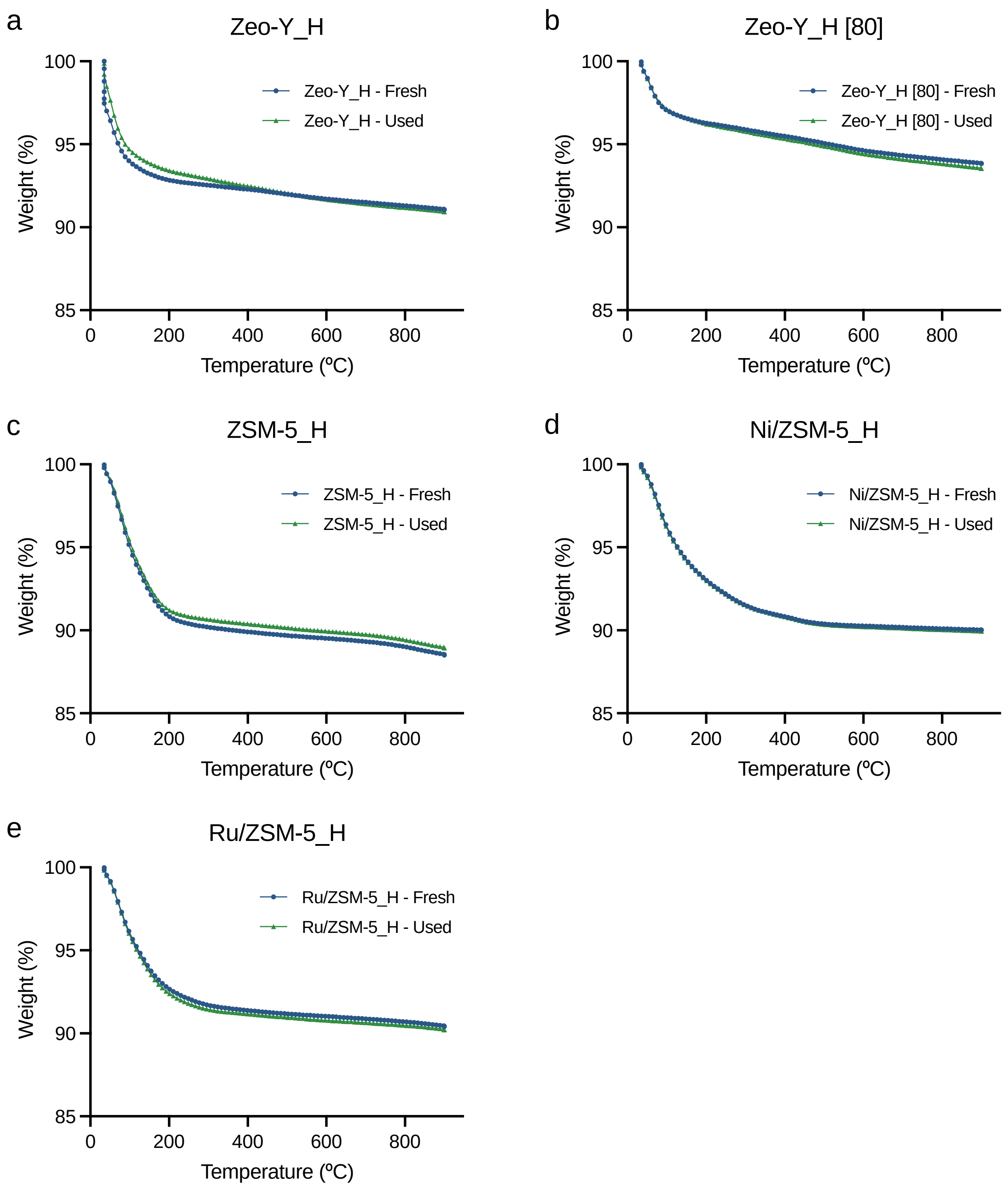

Supplementary Figure 21 TGA analysis of fresh and used catalyst. **a** Zeo-Y_H **b** Zeo-Y_H [80] **c** ZSM-5_H **d** Ni/ZSM-5_H **e** Ru/ZSM-5 _H with a ramping rate of 5 ºC/min from 35 to 900 ºC and a flow rate of 20 mL/min under air. Source data are provided as a Source Data file.

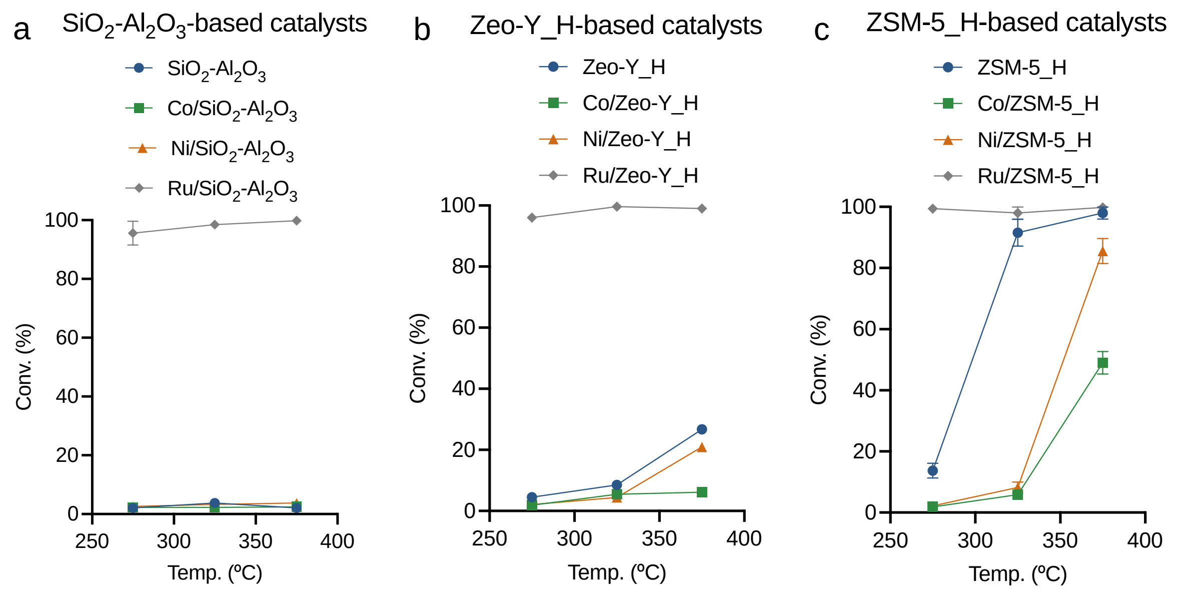

Supplementary Figure 22 nC_{16} (1.59 g) deconstructions with 45 bar H_2 at 275, 325 and 375 ºC, 2 hrs. In the presence of **a** SiO_2-Al_2O_3-based catalysts (0.1 g, metal loading = 2.5 wt%): SiO_2-Al_2O_3, Co/SiO_2-Al_2O_3, Ni/SiO_2-Al_2O_3, Ru/SiO_2-Al_2O_3, **b** Zeo-Y_H-based catalysts (0.1 g, metal loading = 2.5 wt%): Zeo-Y_H, Co/Zeo-Y_H, Ni/Zeo-Y_H, Ru/Zeo-Y_H, **c** ZSM-5_H-based catalysts (0.1 g, metal loading = 2.5 wt%): ZSM-5_H, Co/ZSM-5_H, Ni/ZSM-5_H, Ru/ZSM-5_H. Error bars = standard deviation. Source data are provided as a Source Data file.

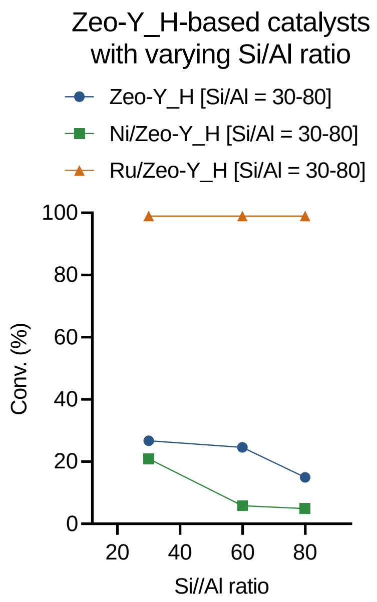

Supplementary Figure 23 nC_{16} (1.59 g) deconstructions with 45 bar H_2 at 375 ºC, 2 hrs. In the presence of Zeo-Y_H-based catalysts with varying Si/Al ratios (0.1 g, metal loading = 2.5 wt%): Zeo-Y_H, Zeo-Y_H [60], Zeo-Y_H [80], Ni/Zeo-Y_H, Ni/Zeo-Y_H [60], Ni/Zeo-Y_H [80], Ru/Zeo-Y_H, Ru/Zeo-Y_H [60], and Ru/Zeo-Y_H [80]. Error bars = standard deviation. Source data are provided as a Source Data file.

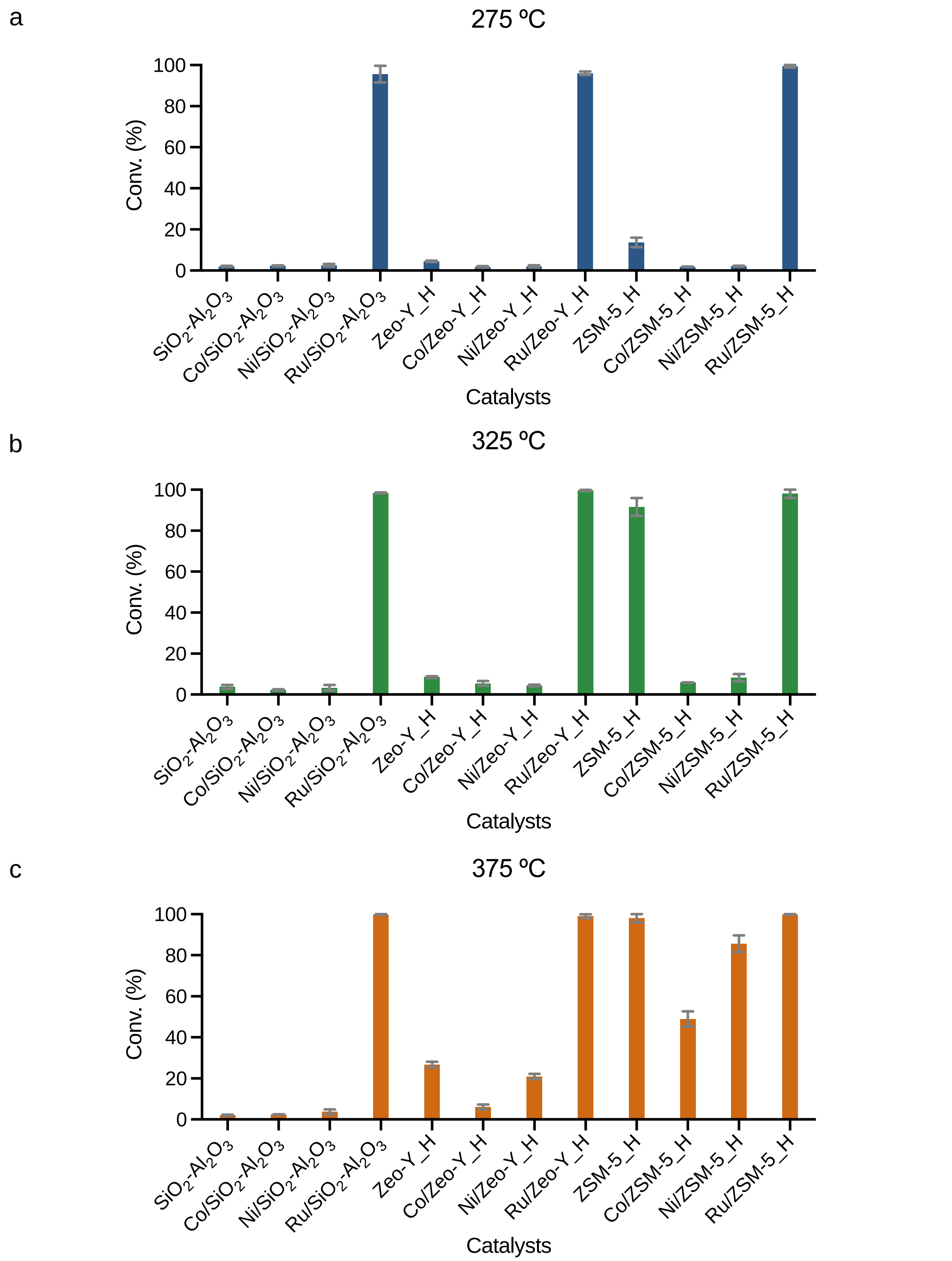

Supplementary Figure 24 nC_{16} (1.59 g) deconstruction with 45 bar H_2, 2 hrs. In the presence of SiO_2-Al_2O_3, Co/SiO_2-Al_2O_3, Ni/SiO_2-Al_2O_3, Ru/SiO_2-Al_2O_3, Zeo-Y_H, Co/Zeo-Y_H, Ni/Zeo-Y_H, Ru/Zeo-Y_H, ZSM-5_H, Co/ZSM-5_H, Ni/ZSM-5_H and Ru/ZSM-5_H (cat. = 0.1 g, metal loading = 2.5 wt%) at **a** 275 ºC, **b** 325 ºC and **c** 375 ºC. Error bars = standard deviation. Source data are provided as a Source Data file.

Supplementary Table 1 XPS surface relative atomic concentration of the ZSM-5_H-based catalysts.

Catalyst	Si% (*2p*)	Al% (*2p*)	O% (*1s*)	Co% (*2p*)	Ni% (*2p*)	Ru% (*3d*)
ZSM-5_H	28.6	2.8	68.7	-	-	-
Co/ZSM-5_H	22.4	0.8	67.0	9.8	-	-
Ni/ZSM-5_H	22.3	4.8	64.6	-	8.3	-
Ru/ZSM-5_H	26.6	3.2	67.6	-	-	2.6

Supplementary Table 2 XPS surface species and their relative concentrations of the ZSM-5_H-based catalysts.

Catalyst	Specie %			
Co/ZSM-5_H	Co(0)% (*2p*)	CoO% (*2p*)	$Co(OH)_2$% (*2p*)	
	1.4	2.2	96.4	
Ni/ZSM-5_H	Ni(0)% (*2p*)	NiO% (*2p*)	Ni(O)OH% (*2p*)	$Ni(OH)_2$% (*2p*)
	1.8	0.3	60.4	37.5
Ru/ZSM-5_H	Ru(0)% (*3d*)	RuO_2% (*3d*)	$RuO_2 \cdot H_2O$% (*3d*)	$RuCl_3$% (*3d*)
	86.0	3.9	6.7	3.4

Supplementary Table 3 *n*-Hexadecane deconstructions with the 12 catalysts from the main library at 275, 325, and 375 ºC including the carbon balance and hydrogen consumption.

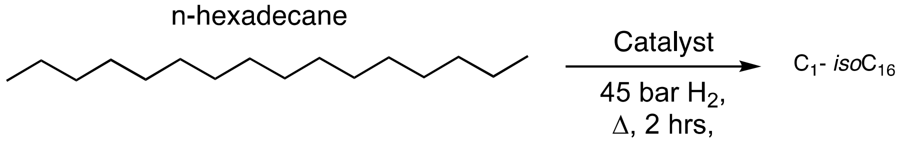

Entry	Catalyst	Temp. (ºC)	Conv. (%) [g]	C_{1-4} Yield (%) [g]	C_{5-16} Yield (%) [g]	H_2 Cons. (%) [g]
1	SiO_2-Al_2O_3	275 ºC	2.1±0.2 [0.03±0.00]	0.0 [0.00]	2.1 [0.03]	1 [0.00]
2	SiO_2-Al_2O_3	325 ºC	3.8±1.0 [0.06±0.02]	0.1 [0.00]	3.7 [0.06]	6 [0.01]
3	SiO_2-Al_2O_3	375 ºC	2.1±0.2 [0.03±0.00]	0.1 [0.00]	2.0 [0.03]	5 [0.01]
4	Zeo-Y_H	275 ºC	4.5±0.3 [0.07±0.00]	0.3 [0.01]	4.2 [0.07]	2 [0.00]
5	Zeo-Y_H	325 ºC	8.5±0.5 [0.14±0.01]	0.7 [0.01]	7.7 [0.12]	10 [0.02]
6	Zeo-Y_H	375 ºC	26.7±1.4 [0.42±0.02]	3.3 [0.05]	23.3 [0.37]	11 [0.03]
7	ZSM-5_H	275 ºC	13.7±2.4 [0.22±0.04]	3.8 [0.06]	10.0 [0.16]	5 [0.01]
8	ZSM-5_H	325 ºC	91.6±4.4 [1.46±0.07]	35.5 [0.58]	56.2 [0.90]	17 [0.04]
9	ZSM-5_H	375 ºC	98.0±2.0 [1.56±0.03]	77.3 [1.27]	20.8 [0.33]	23 [0.06]
10	Co/SiO_2-Al_2O_3	275 ºC	2.3±0.2 [0.04±0.00]	0.1 [0.00]	2.2 [0.03]	4 [0.01]
11	Co/SiO_2-Al_2O_3	325 ºC	2.3±0.3 [0.04±0.00]	0.0 [0.00]	2.2 [0.03]	2 [0.00]
12	Co/SiO_2-Al_2O_3	375 ºC	2.4±0.1 [0.04±0.00]	0.1 [0.00]	2.2 [0.03]	7 [0.02]
13	Co/Zeo-Y_H	275 ºC	1.9±0.4 [0.03±0.01]	0.0 [0.00]	1.9 [0.03]	4 [0.01]
14	Co/Zeo-Y_H	325 ºC	5.5±1.2 [0.09±0.02]	0.2 [0.00]	5.2 [0.08]	6 [0.01]
15	Co/Zeo-Y_H	375 ºC	6.1±1.2 [0.10±0.02]	1.2 [0.02]	4.9 [0.08]	8 [0.02]
16	Co/ZSM-5_H	275 ºC	1.9±0.1 [0.03±0.00]	0.1 [0.00]	1.8 [0.03]	6 [0.01]
17	Co/ZSM-5_H	325 ºC	5.9±0.1 [0.09±0.00]	1.2 [0.02]	4.7 [0.07]	6 [0.01]
18	Co/ZSM-5_H	375 ºC	49.0±3.7 [0.78±0.06]	12.7 [0.21]	36.3 [0.58]	13 [0.03]
19	Ni/SiO_2-Al_2O_3	275 ºC	2.6±0.6 [0.04±0.01]	0.1 [0.00]	2.6 [0.04]	3 [0.01]
20	Ni/SiO_2-Al_2O_3	325 ºC	3.3±1.4 [0.05±0.02]	0.1 [0.00]	3.2 [0.05]	4 [0.01]
21	Ni/SiO_2-Al_2O_3	375 ºC	3.8±1.2 [0.06±0.02]	0.2 [0.00]	3.7 [0.06]	7 [0.02]
22	Ni/Zeo-Y_H	275 ºC	2.1±0.6 [0.03±0.01]	0.6 [0.01]	1.5 [0.02]	4 [0.01]
23	Ni/Zeo-Y_H	325 ºC	4.4±0.4 [0.07±0.01]	0.4 [0.01]	4.0 [0.06]	5 [0.01]
24	Ni/Zeo-Y_H	375 ºC	20.9±1.3 [0.33±0.02]	0.4 [0.01]	20.5 [0.31]	8 [0.02]
25	Ni/ZSM-5_H	275 ºC	2.2±0.2 [0.03±0.00]	0.3 [0.00]	1.9 [0.03]	4 [0.01]
26	Ni/ZSM-5_H	325 ºC	8.2±1.8 [0.13±0.03]	0.5 [0.01]	7.7 [0.12]	7 [0.02]
27	Ni/ZSM-5_H	375 ºC	85.6±4.1 [1.36±0.07]	28.2 [0.46]	57.3 [0.92]	13 [0.03]
28	Ru/SiO_2-Al_2O_3	275 ºC	95.6±4.0 [1.52±0.06]	41.5 [0.71]	54.2 [0.86]	27 [0.07]
29	Ru/SiO_2-Al_2O_3	325 ºC	98.5±0.3 [1.57±0.00]	75.8 [1.33]	22.6 [0.36]	60 [0.14]
30	Ru/SiO_2-Al_2O_3	375 ºC	99.8±0.2 [1.59±0.00]	96.6 [1.72]	3.3 [0.05]	83 [0.20]
31	Ru/Zeo-Y_H	275 ºC	96.0±0.9 [1.53±0.01]	56.6 [0.99]	39.3 [0.63]	44 [0.11]
32	Ru/Zeo-Y_H	325 ºC	99.6±0.3 [1.58±0.00]	91.5 [1.62]	8.3 [0.13]	73 [0.18]
33	Ru/Zeo-Y_H	375 ºC	99.0±1.0 [1.57±0.02]	92.4 [1.65]	6.9 [0.11]	85 [0.21]
34	Ru/ZSM-5_H	275 ºC	99.4±0.6 [1.58±0.01]	92.8 [1.65]	6.6 [0.10]	54 [0.13]
35	Ru/ZSM-5_H	325 ºC	98.0±2.0 [1.56±0.03]	99.7 [1.78]	0.3 [0.01]	88 [0.21]
36	Ru/ZSM-5_H	375 ºC	99.8±0.2 [1.59±0.00]	98.8 [1.77]	1.1 [0.02]	88 [0.21]

Reaction conditions: *n*-hexadecane (1.59 g, 7.0 mmol), catalyst (0.1 g, metal loading = 2.5 wt%), S/C ratio (substrate/catalyst weight ratio) ~16, 45 bar H_2, 2 hrs. * All yields were calculated as the carbon yield and isomerized C_{16} (*iso*C_{16}) are considered as products. Note that ~87% H_2 consumption (~105.0 mmol) is able to produce methane quantitatively due to the ~1.15 eq. H_2 stichometry.

Supplementary Table 4 *n*-Hexadecane deconstruction using the Zeo-Y_H-based catalysts with varying SARs including the carbon balance and hydrogen consumption.

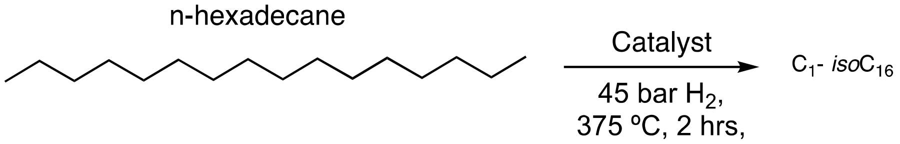

Entry	Catalyst	Conv. (%) [g]	C_{1-4} Yield (%) [g]	C_{5-16} Yield (%) [g]	H_2 Cons. (%) [g]
1	Zeo-Y_H	26.7±1.4 [0.42±0.02]	3.3 [0.05]	23.3 [0.37]	11 [0.03]
2	Zeo-Y_H [60]	24.6±1.2 [0.39±0.02]	3.8 [0.06]	20.8 [0.33]	8 [0.02]
3	Zeo-Y_H [80]	14.9±0.6 [0.24±0.01]	2.0 [0.03]	12.8 [0.20]	6 [0.01]
4	Ni/Zeo-Y_H	20.9±1.3 [0.33±0.02]	0.4 [0.01]	20.5 [0.31]	8 [0.02]
5	Ni/Zeo-Y_H [60]	5.8±0.5 [0.09±0.01]	0.9 [0.01]	4.9 [0.08]	7 [0.02]
6	Ni/Zeo-Y_H [80]	4.9±0.7 [0.08±0.01]	0.6 [0.01]	4.3 [0.07]	4 [0.01]
7	Ru/Zeo-Y_H	99.0±1.0 [1.57±0.02]	92.4 [1.65]	6.9 [0.11]	85 [0.21]
8	Ru/Zeo-Y_H [60]	99.0±1.0 [1.57±0.02]	98.0 [1.76]	2.0 [0.02]	86 [0.21]
9	Ru/Zeo-Y_H [80]	99.0±1.0 [1.57±0.02]	97.2 [1.75]	2.8 [0.03]	87 [0.21]

Reaction conditions: *n*-hexadecane (1.59 g, 7.0 mmol), catalyst (0.1 g, metal loading = 2.5 wt%), S/C ratio (substrate/catalyst weight ratio) ~16, 45 bar H_2, 375 ºC, 2 hrs. * All yields were calculated as the carbon yield and isomerized C_{16} (*iso*C_{16}) are considered as products. Note that ~87% H_2 consumption (~105.0 mmol) is able to produce methane quantitatively due to the ~1.15 eq. H_2 stichometry.

Supplementary Table 5 Degrees of saturation of the liquid products obtained from the deconstruction of *n*-hexadecane.

Entry	Catalyst	Conversion (%)	Saturated (%, δ = 0.25-2.0)	Unsaturated (%, δ = 2.0-6.0)	Aromatic (%, δ = 6.0-8.0)
1	Zeo-Y_H	26.7±1.4	98.8±0.1	1.2±0.1	0.1±0.1
2	Zeo-Y_H [60]	24.6±1.2	98.6±0.1	1.4±0.1	0.1±0.1
3	Zeo-Y_H [80]	14.9±0.6	98.7±0.1	1.3±0.1	0.1±0.1
4	Ni/Zeo-Y_H	20.9±1.3	99.8±0.2	0.2±0.2	0.1±0.1
5	Ni/Zeo-Y_H [60]	5.8±0.5	99.7±0.3	0.3±0.3	0.1±0.1
6	Ni/Zeo-Y_H [80]	4.9±0.7	99.5±0.5	0.5±0.5	0.1±0.1

Reaction conditions: *n*-hexadecane (1.59 g, 7.0 mmol), catalyst (0.1 g, metal loading = 2.5 wt%), 45 bar H_2, 375 ºC, 2 hrs. Note that degrees of saturation are defined by the ratio of proton integrations in the ^1H NMR spectra to indicate the adjacent carbon-carbon bonds (saturated: δ = 0.25-2.0, unsaturated: δ = 2.0-6.0 and aromatics: δ = 2.0-6.0) given the C-H and C-C bond exclusivity of hydrocarbons.

Supplementary References

1. Biesinger, M. C. *et al.* Resolving surface chemical states in XPS analysis of first row transition metals, oxides and hydroxides: Cr, Mn, Fe, Co and Ni. *Appl. Surf. Sci.* **257**, 2717–2730 (2011).
2. Morgan, D. J. Resolving ruthenium: XPS studies of common ruthenium materials. *Surf. Interface Anal.* **47**, 1072–1079 (2015).
